# Body image mediates the relationship between post-surgery needs and health-related quality of life among women with breast cancer: a cross-sectional study

**DOI:** 10.1186/s12955-020-01400-5

**Published:** 2020-06-01

**Authors:** Kaina Zhou, Wen Wang, Minjie Li, Jinghua An, Lanting Huo, Xiaole He, Jin Li, Xiaomei Li

**Affiliations:** School of Nursing, Xi’an Jiaotong University Health Science Centre, No. 76 Yanta West Road, Xi’an, 710061 Shaanxi China

**Keywords:** Breast cancer, Needs, Body image, Health-related quality of life, China

## Abstract

**Background:**

Although body image (BI) disturbance is a common problem that often contributes to poor health-related quality of life (HRQoL) among women with breast cancer following surgery, the mediating role of BI (as a self-perceptive factor) in the relationship between needs and HRQoL after controlling for socio-demographic factors remains unclear. The purpose of this study was to identify the mediating role of BI between post-surgery needs and HRQoL after controlling for socio-demographic factors among women with breast cancer.

**Methods:**

In this cross-sectional study, the primary outcome was HRQoL (as measured with the 36-item Short-Form Health Survey version 2 [SF-36v2] and Functional Assessment of Cancer Therapy-Breast version 4.0 [FACT-Bv4.0]). The secondary outcomes included needs (measured in terms of needs importance [NI] and needs satisfaction [NS]) and BI. Structural equation modeling was used to identify the mediating role of BI between needs and HRQoL while considering socio-demographics.

**Results:**

The 406 eligible patients reported poor HRQoL, and approximately half reported important unmet needs and poor BI. NI, NS, and socio-demographics had differing direct effects on BI and HRQoL, and contrasting indirect effects on HRQoL via BI. NI, NS, surgery type, presence of chronic disease, and BI explained 4% of the variance in the SF-36v2 physical component summary score; NI, NS, surgery type, residence, and BI explained 20% of the variance in the mental component summary score; and NI, NS, marital status, employment status, radiotherapy, and BI explained 33% of the variance in the FACT-Bv4.0 total score.

**Conclusions:**

After surgery, women with breast cancer have poor HRQoL and BI, and important unmet needs. BI mediates the relationship between needs and HRQoL after controlling for socio-demographics. The present findings provide information for developing comprehensive BI-based needs interventions and preparing targeted health-management programs for patients with breast cancer.

## Background

As surgery is the primary modality for breast cancer treatment, the majority of post-surgery patients experience body image (BI; i.e., one’s perspective of his/her own body) disturbance and impaired health-related quality of life (HRQoL), which might subsequently require changes in one’s capacity to cope with the illness [1, 2]. According to Doyal and Gough, a need is a lack of something that is required or desired, and unsatisfied needs have been known to cause psychological distress [3]. However, due to varying socio-demographic characteristics and needs, patients might react differently to BI disturbances and experience different levels of HRQoL impairment [4]. Thus, the change in patients’ needs during the disease course is a critical issue that requires the attention of health care providers. Given that socio-demographic features can be difficult to change, it is more critical to focus on needs (particularly their importance and satisfaction) for such patients in order to provide further information for the development of relevant interventions.

Although BI disturbance is a common problem that often induces poor HRQoL among women with breast cancer following surgery [5], the mediating role of BI (as a self-perceptive factor) in the relationship between needs and HRQoL (after controlling for socio-demographic characteristics) remains unclear. Since a positive BI and met needs are related to improved HRQoL [6], it is worthwhile to identify the influencing path between needs and HRQoL via BI. If BI does play a mediating role, needs-oriented interventions that consider BI might be used for post-surgery health improvement among women with breast cancer.

We, therefore, aimed to clarify the mediating role of BI in the relationship between post-surgery needs (i.e., their importance and satisfaction) and HRQoL, after controlling for the influence of potentially related socio-demographic factors among women with breast cancer. Two hypotheses were developed: (a) patients with different needs and socio-demographics would experience varying levels of BI and HRQoL impairment, and (b) BI would mediate the relationship between needs and HRQoL after controlling for the influence of socio-demographic characteristics. Our findings should provide valuable information for planning target intervention programs regarding health and BI management among this population.

## Methods

### Study design

A cross-sectional approach was adopted.

### Participants and data collection

All participants were women with breast cancer who had been admitted to one of two hospitals in Xi’an following surgery. The patients were recruited via convenience sampling. Patients who were 18 years or above and spoke Chinese were included. However, patients who had other breast diseases, cognitive disorders (which were screened by a psychiatrist based on criteria of the Diagnostic and Statistical Manual of Mental Disorders, 5th ed.), or other chronic diseases (e.g., endocrine, cardiovascular, pneumonia, or infectious disease), and refused to provide written informed consent were excluded.

To ensure this cross-sectional study was impactful, we estimated the required sample size using the metric of 5 to 10 participants per item in the questionnaire survey [7]. Of the questionnaires used in the study, the instruments with the most items (i.e., 36 items) were the 36-item Short-Form Health Survey version 2 (SF-36v2) and the Functional Assessment of Cancer Therapy-Breast version 4.0 (FACT-Bv4.0). Thus, the estimated sample size was 180 to 360 patients. However, anticipating sample loss, we enlarged the estimated sample size by 20% [7]. Consequently, the final sample size estimation was 216 to 432 participants.

Data collection was conducted from March to October 2018. The questionnaires were completed by the recruited patients within 6 months after surgery; patients with reading or writing difficulties were interviewed by trained data collectors who recorded the patients’ responses.

### Measurements

The primary outcome was HRQoL, which was measured using the SF-36v2 (a generic instrument) and the FACT-Bv4.0 (a disease-specific instrument). The secondary outcomes included BI, which was assessed using the Body Image Self-Rating Questionnaire for Breast Cancer (BISQ-BC), and needs, which were measured with the Needs Self-Rating Questionnaire for Breast Cancer (NSQ-BC). In this study, BI was considered as a potential mediating variable in the relationship between needs and HRQoL.

#### SF-36v2

The Chinese 36-item SF-36v2 was provided by QualityMetric Incorporated [8]. It comprises eight scales: physical function (PF), role-physical (RP), bodily pain (BP), general health (GH), vitality (VT), social function (SF), role-emotional (RE), and mental health (MH). These, in turn, form two summary components: the physical component summary (PCS) and the mental component summary (MCS). All scores were calculated using Health Outcomes Scoring Software 2.0 (QualityMetric Incorporated) based on norms, with a mean of 50 and a standard deviation of 10 [9]. For all scales and summary components, higher scores indicated better HRQoL. For this study, the Cronbach’s α was 0.91.

#### FACT-Bv4.0

The 36-item Chinese FACT-Bv4.0 includes a general subscale (FACT-G) consisting of physical well-being (PWB), social/family well-being (SWB), emotional well-being (EWB), and functional well-being (FWB), and a subscale measuring breast-cancer-specific additional concerns (BCS) [10]. Each item was rated using a five-point Likert scale (from 0 to 4), with a total score ranging from 0 to 144. A higher score indicated better HRQoL [11]. The Chinese FACT-Bv4.0 has been found to have satisfactory psychometrics among breast cancer patient populations [10]. For this study, the Cronbach’s α was 0.88.

#### BISQ-BC

The 33-item BISQ-BC was developed by the authors, including subscales on BI-related self-cognition (BI-SCo), BI-related behavior change (BI-BC), BI-related arm change (BI-AC), BI-related sexual activity change (BI-SAC), BI-related role change (BI-RC), BI-related psychological change (BI-PC), and BI-related social change (BI-SC) [12]. Each item was rated using a five-point Likert scale (1 = *strongly disagree* to 5 = *strongly agree*). All scores were standardized using the following formula: ([actual score – the lowest possible score]/ [the highest possible score – the lowest possible score]) × 100 [13]. All standardized scores ranged from 0 to 100, with higher scores representing worse BI. For this study, the Cronbach’s α was 0.86.

#### NSQ-BC

The authors also developed the 28-item NSQ-BC based on a review of existing empirical literature regarding the needs of Chinese women with breast cancer, which examined their physical needs (PHY), psychological needs (PSY), respect/self-esteem needs (RSE), information needs (INF), and rehabilitation needs (REH) [14]. Based on the needs assessment and intervention outcome evaluation, the authors divided the NSQ-BC into the following dimensions: Needs Importance (NI; for needs assessment, i.e., whether a given need is the most needed or desired one) and Needs Satisfaction (NS; for intervention outcome evaluation; i.e., whether a given need has been fully met) dimensions for this questionnaire. For a more detailed understanding of the NSQ-BC, please see a previously published study [14]. Each item was rated using a five-point Likert format (1 = *not important/satisfied* to 5 = *very important/satisfied*). The scale and total scores were standardized using the same formula as the BISQ-BC [13]. All standardized scores ranged from 0 to 100, with higher scores indicating higher levels of NI and NS. For this study, the Cronbach’s α was 0.73 (NI) and 0.71 (NS).

### Data analyses

An independent samples *t*-test was applied to compare BI and HRQoL between patients with different levels of NI and NS, and to compare HRQoL among patients with different levels of BI disturbance. Meanwhile, a one-sample *t*-test was used to compare patients’ SF-36v2 scores with the norm score (mean = 50). A multiple linear stepwise regression analysis was performed to identify the significant influencing factors of BI and HRQoL.

Structural Equation Modeling (SEM) is a method for building, estimating, and testing theoretical models of the relationships between variables. It can be used in lieu of multiple regression and other methods to analyze the strength of the correlations between individual variable indicators within a given population [15]. In this study, the previously determined significant factors were used in SEM to identify the mediating role of BI on the relationship between NI, NS, socio-demographic factors, and HRQoL. Standardized direct, indirect, and total effects with corresponding 95% bias-corrected confidence intervals were measured using the bootstrapping method [15, 16].

The mediating effect was examined by determining whether (i) the independent variables had significant direct effects on the mediator (i.e., the factor with mediating roles between certain variables); (ii) the independent variables had significant direct effects on the outcome variable; and (iii) the independent variables had significant indirect effects, and the mediator had significant direct effects, on the outcome variable [17].

The model fit was tested using the normed chi-square (NC; desired value< 2.0), χ^2^ value (desired significance *P* > 0.05), goodness-of-fit index (GFI; desired value> 0.90), adjusted GFI (AGFI; desired value> 0.90), and root mean square error of approximation (RMSEA; desired value< 0.08) [15].

The database was built using Epidata 3.1. Two data managers double-entered the data to minimize the risk of data-entry errors. The data were analyzed using SPSS Statistics 23.0 and AMOS 23.0, and values of *P* < 0.05 (two-tailed) were considered statistically significant.

### Ethics statement

The study protocol was approved by the Biomedical Ethics Committee of Xi’an Jiaotong University Health Science Center (Reference No.: 2015–170). Informed consent was obtained from all participants in the study.

## Results

Among the 430 recruited patients, 406 (94.4%) completed the questionnaire survey. Of the 24 excluded patients, 15 had a history of chronic medical disorders, and 9 refused to provide written informed consent. Table 1 shows the detailed socio-demographic characteristics of the participants.

**Table 1:** Patient characteristics (*N* = 406)

Characteristics	***n*** (%)
**Socio-demographics**	
Age (years) (mean ± SD) (range: 22–75)	49.77 ± 9.57
Education level	
Primary and lower	84 (20.7)
Secondary	234 (57.6)
Tertiary	88 (21.7)
Marital status	
Married	387 (95.3)
Other	19 (4.7)
Employment status	
Employed	152 (37.4)
Unemployed	160 (39.4)
Retired	94 (23.2)
Average monthly income over the past year (Chinese yuan)	
< 1000	100 (24.6)
1000–3000	174 (42.9)
> 3000	132 (32.5)
Residence	
Rural	200 (49.3)
Urban	206 (50.7)
Suffering from chronic disease(s)	
Yes	80 (19.7)
No	326 (80.3)
**Clinical characteristics**	
Illness stage	
I	74 (18.2)
II	211 (52.0)
III	95 (23.4)
IV	26 (6.4)
Surgery type	
Modified radical mastectomy	258 (63.5)
Total mastectomy	99 (24.4)
Lumpectomy and axillary dissection	37 (9.1)
Breast conserving surgery	12 (3.0)
Chemotherapy	
Yes	402 (99.0)
No	4 (1.0)
Radiotherapy	
Yes	46 (11.3)
No	360 (88.7)
Endocrinotherapy	
Yes	34 (8.4)
No	372 (91.6)
**Needs importance (NI) (mean ± SD)**	
Respect/self-esteem needs (RSE)	76.38 ± 12.44
Rehabilitation needs (REH)	73.61 ± 13.57
Information needs (INF)	71.54 ± 13.74
Physical needs (PHY)	65.83 ± 17.84
Psychological needs (PSY)	58.43 ± 19.58
**Needs satisfaction (NS) (mean ± SD)**	
Respect/self-esteem needs (RSE)	69.73 ± 12.49
Rehabilitation needs (REH)	59.30 ± 16.09
Physical needs (PHY)	55.99 ± 17.24
Information needs (INF)	52.66 ± 16.84
Psychological needs (PSY)	51.44 ± 18.22
**Body image (BI) (mean ± SD)**	
BI-related behavior change (BI-BC)	62.76 ± 14.83
BI-related self-cognition change (BI-SCo)	59.04 ± 13.33
BI-related arm change (BI-AC)	58.50 ± 11.93
BI-related psychological change (BI-PC)	53.76 ± 16.72
BI-related sexual activity change (BI-SAC)	52.83 ± 16.18
BI-related social change (BI-SC)	52.03 ± 23.66
BI-related role change (BI-RC)	50.03 ± 17.18
**36-item Short Form Health Survey version 2.0 (SF-36v2) (mean ± SD)**	
Physical component summary (PCS)	43.72 ± 6.15
Mental component summary (MCS)	42.23 ± 9.09
Physical functioning (PF)	45.54 ± 6.51
Role-physical (RP)	34.85 ± 9.69
Bodily pain (BP)	47.53 ± 9.89
General Health (GH)	42.72 ± 8.89
Vitality (VT)	48.75 ± 8.36
Social functioning (SF)	39.15 ± 10.53
Role-emotional (RE)	38.59 ± 10.70
Mental health (MH)	44.12 ± 8.25
**Functional Assessment of Cancer Therapy-Breast version 4.0 (FACT-Bv4.0) (mean ± SD)**	
Physical well-being (PWB)	17.72 ± 5.07
Social/family well-being (SWB)	18.46 ± 4.75
Emotional well-being (EWB)	17.10 ± 4.34
Functional well-being (FWB)	13.59 ± 5.42
Breast cancer specific additional concerns (BCS)	21.84 ± 5.09
Total score	88.71 ± 17.25

*SD* standard deviation

### NI, NS, BI, and HRQoL

The mean NI score was 68.87 ± 12.66 (range: 23–100); 46.6% (*n* = 189) of the patients considered their needs as important (median score > 69). The mean NS score was 56.99 ± 14.11 (range: 22–100); 50% (*n* = 203) of the patients considered their needs to be unsatisfied (median score ≤ 55). The respect/self-esteem needs scale score was highest for both the NI and NS dimensions (Table 1).

The total mean BI score was 55.84 ± 11.13 (range: 14–90); 52.5% (*n* = 213) of the patients reported poor BI (median score > 56). The BI-related behavior change scale (mean = 62.76 ± 14.83) had the highest score (Table 1).

**Fig. 1:**
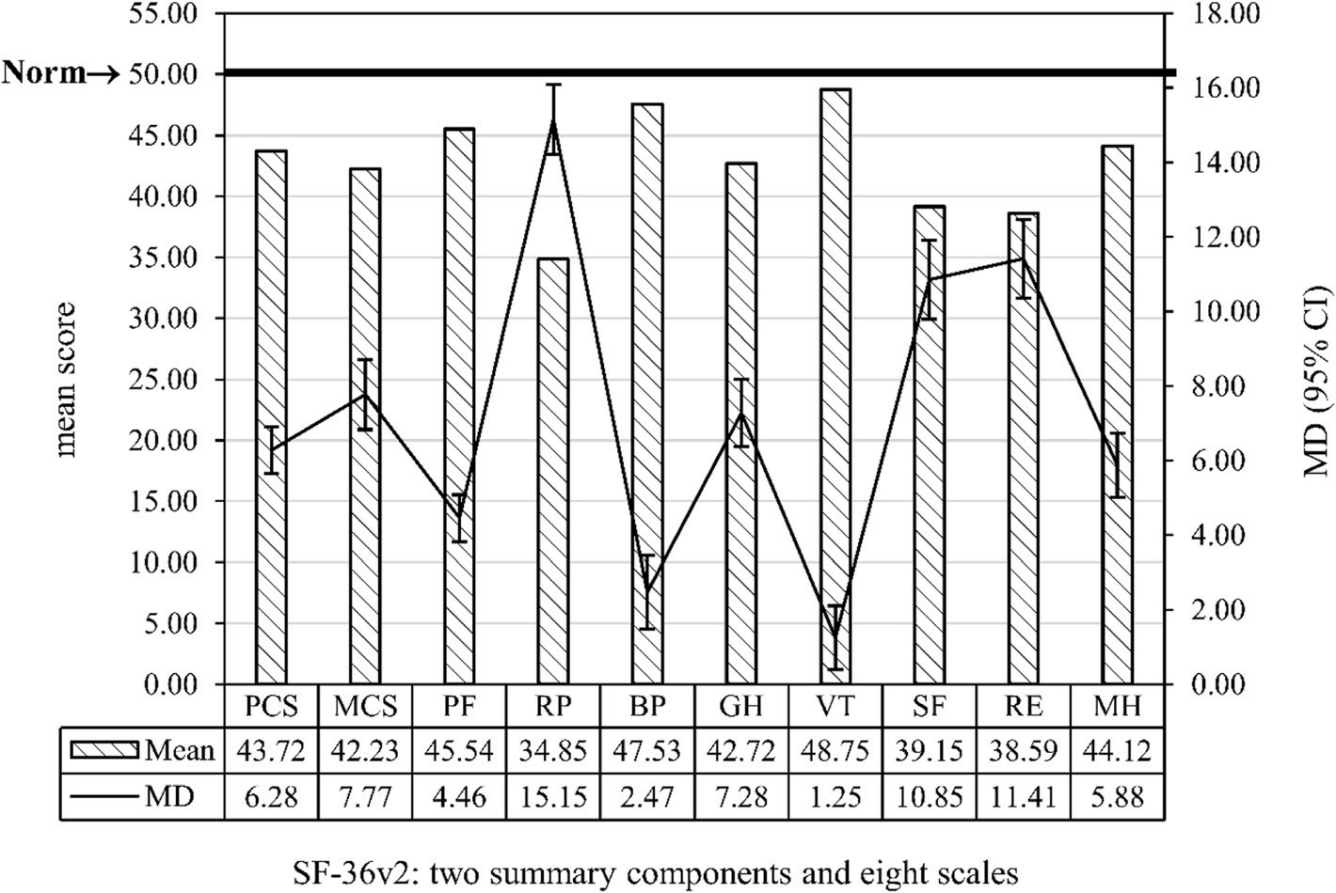
Scores on the two summary components and eight scales of the SF-36v2 Health Survey. MD: mean difference between the scores and the norm with 95% confidence intervals (CI); SF-36v2: Short-Form 36 Health Survey version 2; PCS: physical component summary; MCS: mental component summary; PF: physical functioning; RP: role-physical; BP: bodily pain; GH: general health; VT: vitality; SF: social functioning; RE: role-physical; MH: mental health

Regarding the SF-36v2, the two summary components and the eight scale scores were all lower than the normed score (*P* < 0.05). The overall psychological health (mean difference in MCS: 7.77) was lower than the overall physical health (mean difference in PCS: 6.28; Fig. 1). The mean FACT-Bv4.0 total score was 88.71 ± 17.25 (range: 33–127). The BCS score (mean = 22.27 ± 5.04) was the highest among the FACT-Bv4.0 subscales (Table 1).

### NI, NS, and socio-demographics concerning BI and HRQoL

Patients with lower NI scores (i.e., median score ≤ 69) had higher scores on the SF-36v2 and FACT-Bv4.0 (*P* < 0.05), while patients with lower NS scores (i.e., median score ≤ 55) had higher scores on the BISQ-BC and lower scores on the SF-36v2 and FACT-Bv4.0 (*P* < 0.05). Moreover, patients with lower BISQ-BC scores (i.e., median score ≤ 56) had higher scores on the SF-36v2 and FACT-Bv4.0 (*P* < 0.05; Table 2).

**Table 2:** Mean differences in BI, SF-36v2, and FACT-Bv4.0 according to NI, NS, and BI scores (N = 406)

	Needs importance (NI) (≤69 vs. > 69)		Needs satisfaction (NS) (≤55 vs. > 55)		BI (≤56 vs. > 56)	
	MD (95%CI)	*P*	MD (95%CI)	*P*	MD (95%CI)	*P*
**Body image (BI)**						
Self-cognition (SCO)	−2.09 (−4.69, 0.52)	0.12	−1.45 (−4.05, 1.15)	0.28	–	–
Behavior change (BC)	−2.48 (−5.37, 0.42)	0.09	3.13 (0.25, 6.01)	**0.03**	–	–
Arm change (AC)	−0.68 (−3.02, 1.65)	0.57	−2.05 (− 4.37, 0.27)	0.08	–	–
Sexual activity change (SAC)	−2.19 (−5.35, 0.98)	0.18	6.84 (3.75, 9.93)	**< 0.001**	–	–
Role change (RC)	−2.54 (−5.90, 0.81)	0.14	3.80 (0.46, 7.13)	**0.026**	–	–
Psychological change (PC)	−1.44 (−4.71, 1.83)	0.39	9.32 (6.19, 12.46)	**< 0.001**	–	–
Social change (SC)	−2.14 (−6.77, 2.49)	0.37	7.39 (2.82, 11.96)	**0.002**	–	–
Body image total score	−2.00 (−4.17, 0.17)	0.07	4.25 (2.11, 6.38)	**< 0.001**	–	–
**SF-36v2**						
Physical function (PF)	0.97 (−0.29, 2.23)	0.13	1.14 (− 0.11, 2.39)	0.07	1.19 (− 0.07, 2.44)	0.06
Role-physical (RP)	1.30 (−0.54, 3.15)	0.17	0.85 (−0.99, 2.70)	0.36	4.22 (2.42, 6.02)	**< 0.001**
Bodily pain (BP)	4.18 (2.24, 6.12)	**< 0.001**	1.78 (−0.19, 3.75)	0.08	0.74 (−1.24, 2.72)	0.46
General health (GH)	0.90 (−0.93, 2.72)	0.33	−2.06 (−3.88, − 0.25)	**0.026**	5.28 (3.53, 7.03)	**< 0.001**
Vitality (VT)	−0.88 (−2.60, 0.85)	0.32	−2.75 (−4.45, −1.05)	**0.002**	4.02 (2.34, 5.70)	**< 0.001**
Social function (SF)	3.19 (1.08, 5.31)	**0.003**	1.36 (−0.77, 3.49)	0.21	4.86 (2.78, 6.95)	**< 0.001**
Role-emotional (RE)	2.88 (0.77, 4.99)	**0.007**	−0.10 (−2.26, 2.02)	0.92	5.90 (3.85, 7.94)	**< 0.001**
Mental health (MH)	1.44 (− 0.29, 3.17)	0.10	−2.80 (−4.51, −1.09)	**0.001**	5.52 (3.87, 7.17)	**< 0.001**
Physical component summary (PCS)	1.50 (0.23, 2.77)	**0.021**	−1.38 (−2.65, −0.11)	**0.033**	1.27 (−0.005, 2.53)	0.05
Mental component summary (MCS)	1.80 (− 0.09, 3.68)	0.06	−2.19 (−4.07, − 0.32)	**0.022**	6.55 (4.77, 8.33)	**< 0.001**
**FACT-Bv4.0**						
Physical well-being (PWB)	0.64 (−0.36, 1.64)	0.21	−1.05 (−2.04, − 0.06)	**0.038**	3.17 (2.22, 4.12)	**< 0.001**
Social/family well-being (SWB)	−2.11 (−3.43, − 0.80)	**0.002**	− 2.53 (− 3.84, − 1.23)	**< 0.001**	1.59 (0.26, 2.92)	**0.019**
Emotional well-being (EWB)	0.66 (− 0.21, 1.53)	0.14	− 2.35 (− 3.19, − 1.51)	**< 0.001**	3.52 (2.71, 4.32)	**< 0.001**
Functional well-being (FWB)	−1.27 (− 2.31, − 0.24)	**0.016**	− 2.36 (− 3.38, − 1.35)	**< 0.001**	2.39 (1.38, 3.40)	**< 0.001**
Additional breast cancer-specific concerns (BCS)	1.81 (0.83, 2.79)	**< 0.001**	− 0.94 (− 1.92, 0.05)	0.06	4.21 (3.30, 5.11)	**< 0.001**
FACT-Bv4.0 total score	−2.10 (− 6.99, 2.78)	0.40	−9.90 (− 14.61, −5.20)	**< 0.001**	12.86 (8.32, 17.40)	**< 0.001**

Significant results are presented in bold. Needs importance, needs satisfaction, and BI were grouped by the corresponding median score

“-” signifies no value

*MD* mean difference, *95% CI* 95% confidence interval

Since BI (BISQ-BC total score) is the mediator, it was regarded as either the independent or dependent variable. The findings showed that NI and NS had significant influences on BI and HRQoL, while BI had a significant influence on HRQoL (Table 3). Therefore, BI could be considered a mediator between NI/NS and HRQoL.

**Table 3:** Predictors of body image, PCS, MCS, and FACT-Bv4.0 scores: multiple linear stepwise regression analysis^†^ (*N* = 406)

Dependent variable	Independent variables	B (95%CI)	*P*	VIF^†^
Body image^a^	Needs satisfaction	− 0.34 (− 0.36, − 0.18)	< 0.001	1.02
	Needs importance	0.30 (0.17, 0.36)	< 0.001	1.01
	Lumpectomy and axillary dissection (ref. modified radical mastectomy)	−0.11 (− 7.80, − 0.54)	0.025	1.06
PCS^b^	Body image	−0.12 (− 0.13, − 0.01)	0.019	1.05
	Needs importance	−0.11 (− 0.10, − 0.007)	0.026	1.03
	Chronic disease (ref. yes)	0.10 (0.03, 3.21)	0.045	1.03
MCS^c^	Body image	−0.40 (− 0.42, − 0.27)	< 0.001	1.02
	Residence (ref. rural)	0.14 (1.03, 4.49)	0.002	1.02
	Lumpectomy and axillary dissection (ref. modified radical mastectomy)	−0.10 (−6.49, − 0.50)	0.022	1.05
FACT-Bv4.0^d^	Body image	−0.42 (− 0.80, − 0.44)	< 0.001	1.03
	Unemployed (ref. employed)	−0.21 (−11.68, −3.45)	< 0.001	1.05
	Needs satisfaction	0.16 (0.06, 0.37)	0.008	1.02
	Radiotherapy (ref. yes)	0.13 (0.69, 12.06)	0.028	1.05
	Marital status (ref. married)	−0.13 (−22.80, −1.02)	0.032	1.05

Multiple linear stepwise regression analysis was performed after controlling for the following dummy variables: education level (ref. primary and below), marital status (ref. married), employment status (ref. employed), average monthly income over the past year (Chinese yuan, ref. < 1000), residence (ref. rural), chronic disease (ref. yes), illness stage (ref. 0-I), surgery type (ref. modified radical mastectomy), chemotherapy (ref. yes), radiotherapy (ref. yes), and endocrinotherapy (ref. yes), as well as continuous characteristics (age, body image, psychosocial needs importance, and psychosocial needs satisfaction)

^a^Body image predictor model: R^2^ = 0.11, *F* = 15.72, *P* < 0.001

^b^PCS predictor model: R^2^ = 0.04, *F* = 5.23, *P* = 0.001

^c^MCS predictor model: R^2^ = 0.19, *F* = 30.97, *P* < 0.001

^d^FACT-Bv4.0 predictor model: R^2^ = 0.35, *F* = 20.37, *P* < 0.001

^†^ VIF < 10 indicates no significant multicollinearity

95%CI: 95% confidence interval. VIF: variance inflation factor

**Fig. 2:**
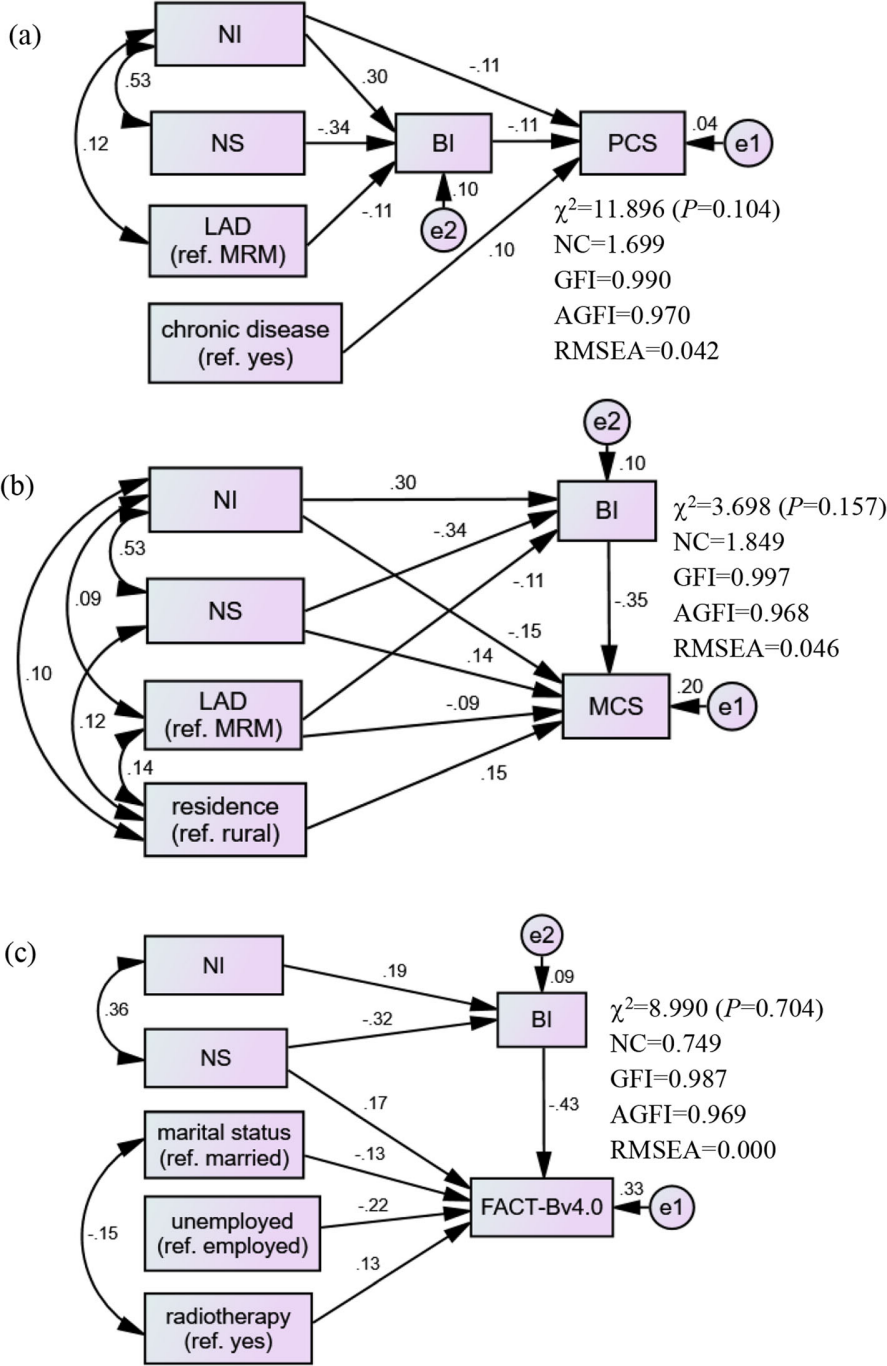
Direct, indirect, and total effects of NI and NS on BI and HRQoL. NC=Normed Chi-square, < 2.0; GFI = goodness-of-fit index, > 0.90; AGFI = adjusted goodness-of-fit index, > 0.90; RMSEA = root mean square error of approximation, < 0.08. LAD: lumpectomy and axillary dissection. MRM: modified radical mastectomy

In the PCS, MCS, and FACT-Bv4.0 models, NI, NS, and socio-demographics had significant direct effects on BI or HRQoL, and significant indirect effects on HRQoL via BI. As for total effects, NI, NS, marital status, employment status, radiotherapy, and BI explained the most variance (33%) in HRQoL (FACT-Bv4.0; Tables 4 and 5, Fig. 2).

**Table 4:** Factors with standardized total/direct/indirect effects on BI, PCS, and MCS (*N* = 406)

Predictor effects	Body image (BI, mediator)		Physical component summary (PCS)		Mental component summary (MCS)	
	B (95%CI)	*P*	B (95%CI)	*P*	B (95%CI)	*P*
**Total effects**						
Body image	–	–	− 0.115 (− 0.202, − 0.003)	0.047	− 0.351 (− 0.434, − 0.258)	0.002
Needs importance	0.297 (0.195, 0.397)	0.002	−0.145 (− 0.245, − 0.043)	0.009	−0.254 (− 0.363, − 0.136)	0.003
Needs satisfaction	−0.341 (− 0.455, − 0.232)	0.002	0.039 (0.004, 0.076)	0.034	0.261 (0.149, 0.373)	0.003
LAD (ref. MRM)	−0.106 (− 0.214, − 0.004)	0.043	0.012 (0.000, 0.036)	0.048	− 0.052 (− 0.155, 0.049)	0.314
Chronic disease (ref. yes)	–	–	0.100 (0.004, 0.206)	0.040	–	–
Residence (ref. rural)	–	–	–	–	0.150 (0.050, 0.236)	0.002
**Direct effects**						
Body image	–	–	− 0.115 (− 0.202, − 0.003)	0.047	− 0.351 (− 0.434, − 0.258)	0.002
Needs importance	0.297 (0.195, 0.397)	0.002	− 0.111 (− 0.211, − 0.009)	0.035	−0.150 (− 0.249, − 0.037)	0.004
Needs satisfaction	− 0.341 (− 0.455, − 0.232)	0.002	–	–	0.142 (0.039, 0.244)	0.007
LAD (ref. MRM)	− 0.106 (− 0.214, − 0.004)	0.043	–	–	− 0.089 (− 0.184, − 0.002)	0.043
Chronic disease (ref. yes)	–	–	0.100 (0.004, 0.206)	0.040	–	–
Residence (ref. rural)	–	–	–	–	0.150 (0.050, 0.236)	0.002
**Indirect effects**						
Body image	–	–	–	–	–	–
Needs importance	–	–	−0.034 (− 0.066, − 0.004)	0.032	− 0.104 (− 0.150, − 0.065)	0.001
Needs satisfaction	–	–	0.039 (0.004, 0.076)	0.034	0.120 (0.077, 0.173)	0.001
LAD (ref. MRM)	–	–	0.012 (0.000, 0.036)	0.048	0.037 (0.003, 0.080)	0.036
Chronic disease (ref. yes)	–	–	–	–	–	–
Residence (ref. rural)	–	–	–	–	–	–

“-” signifies no value

*LAD* Lumpectomy and axillary dissection, *MRM* modified radical mastectomy, *95%CI* 95% confidence interval

**Table 5:** Factors with total, direct, and indirect effects on self-rated body image and FACT-Bv4.0: path analysis (*N*=406)

Predictor effects	Body image (mediator)		FACT-Bv4.0	
	B (95%CI)	*P*	B (95%CI)	*P*
**Total effects**				
Body image	-	-	-0.432 (-0.536, -0.324)	0.001
Needs importance	0.192 (0.044, 0.315)	0.013	-0.083 (-0.143, -0.022)	0.008
Needs satisfaction	-0.316 (-0.470, -0.146)	0.002	0.312 (0.171, 0.436)	0.002
Marital status (ref. married)	-	-	-0.132 (-0.155, -0.009)	0.032
Unemployed (ref. employed)	-	-	-0.223 (-0.371, -0.126)	0.020
Radiotherapy (ref. yes)	-	-	0.130 (0.098, 0.257)	0.041
**Direct effects**				
Body image	-	-	-0.432 (-0.536, -0.324)	0.001
Needs importance	0.192 (0.044, 0.315)	0.013	-	-
Needs satisfaction	-0.316 (-0.470, -0.146)	0.002	0.176 (0.055, 0.300)	0.005
Marital status (ref. married)	-	-	-0.132 (-0.155, -0.009)	0.032
Unemployed (ref. employed)	-	-	-0.223 (-0.371, -0.126)	0.020
Radiotherapy (ref. yes)	-	-	0.130 (0.098, 0.257)	0.041
**Indirect effects**				
Body image	-	-	-	-
Needs importance	-	-	-0.083 (-0.143, -0.022)	0.008
Needs satisfaction	-	-	0.136 (0.060, 0.220)	0.001
Marital status (ref. married)	-	-	-	-
Unemployed (ref. employed)	-	-	-	-
Radiotherapy (ref. yes)	-	-	-	-

“-” signifies no value

95%CI: 95% confidence interval

## Discussion

Interestingly, the needs ordering (in terms of NI and NS) regarding the patients were not very consistent. The two primary needs (i.e., respect/self-esteem and rehabilitation needs) indicate that patients received requisite acceptance and care from health professionals and family members. However, the most commonly important unsatisfied need was information, further indicating that information is one of the most critical support sources during treatment and rehabilitation [18–20]. It suggests that improving access to information must be seriously considered via target interventions. Physical needs were the fourth in NI, but third in NS, which is probably due to improvements in treatment modalities and post-operative nursing programs. The final psychology need reflects that patients were probably neglecting their psychological needs. Since unmet psychological needs are commonly reported [21], it is crucial to focus on patients’ psychological health, enhance professional mental health care, and facilitate patients’ self-care abilities.

Regarding BI-related changes (i.e., behavior, self-cognition, arm, psychological, sexual activity, social activity, and roles) in the BISQ-BC, the first three were seriously affected, and thus require careful observation following surgery. The remaining four should also be seriously assessed while planning programs for improving BI [22].

We found that NI probably does not affect BI disturbance. However, unmet needs are related to poorer BI. Previous studies have reported the influence of psychological distress, socio-demographics, and clinical factors on BI [4]; however, the influence of unmet needs on BI has rarely been explored. This means that while improving patients’ BI, the implementation of programs that enhance NS should also be carefully considered.

The present findings revealed that patients with less NI, more NS, and better BI have better HRQoL [1, 5, 21]. Thus, it appears critical to detect and meet patients’ important needs following surgery; furthermore, BI-related changes should be carefully assessed to plan target intervention programs for improving BI, which would be beneficial for enhancing HRQoL.

Some socio-demographic factors were related to BI and HRQoL, suggesting that for BI and HRQoL care, treatment modalities (e.g., surgery type and adjuvant therapy), basic diseases (e.g., chronic diseases), and life status (e.g., residence, marital status, and employment status) should be seriously considered when planning individualized intervention programs [23, 24]. Due to worry about the cancer recurrence, most Chinese mainland women with breast cancer would like to receive mastectomy, only a small proportion of patients would like to receive breast conservation surgery. Thus, whether breast conservation was associated with improved body image and quality of life in Chinese mainland women with breast cancer needs further study.

In the SEM analysis, BI was a significant mediator of the relationship between NI/NS and HRQoL. Additionally, BI was also a significant mediator between surgery type and HRQoL (measured by the SF-36v2), which further implies that BI should be regarded as an essential interventional factor when improving patients’ health following surgery [5]. This finding supports the notion that BI could be affected by important unmet needs and undergoing radical mastectomy, which could lead to HRQoL impairments [1, 21]. Therefore, post-surgery NI and NS should be regarded as important factors during the assessment and nursing-care process among women with breast cancer, especially those with BI disturbance.

From the treatment viewpoint, the benefits of using breast conservation surgery and breast reconstruction surgery, if permitted, should be provided to the patients, so that the two treatments could be more commonly pursued. Chemotherapy and endocrine therapy were not found to have significant effects on body image, which is probably due to only a small proportion of the patients who did not receive chemotherapy (*n* = 4, 1%), or receive endocrine therapy (*n* = 34, 8.4%). Thus, the explicit assumption that poor body image was the result of chemotherapy and endocrine therapy needs further examination.

In the SF-36v2, patients had significantly lower scores on the two summary components and the eight scales, reflecting poorer HRQoL than what has been observed in the general population. Specifically, the FACT-Bv4.0 scores revealed that patients had few additional concerns in relation to breast cancer, but patients’ functional, emotional, physical, and social/family well-being were affected by the disease. These findings suggest the need for further efforts to improve HRQoL [24].

This study has several limitations. First, the needs, BI, and HRQoL data were based on self-reports and lacked objective indicators. Second, the missing data on post-surgery complications should be considered in our future study, since complications are potential influencing factors for HRQoL. Third, the subjective nature of the survey measures could have generated response biases. Hence, a prospective study may be required to appropriately explore the complex relationship between needs, BI, and HRQoL. Fourth, the cross-sectional design makes it impossible to state the temporality between the exposure and outcome variables. Finally, this study was only conducted in Xi’an, limiting the generalizability of our results among other patient samples.

## Conclusions

The present findings provide information for developing comprehensive BI-based needs interventions and preparing targeted health-management programs for breast cancer patients. A comprehensive intervention considering needs, BI, and socio-demographics (e.g., an individualized multimodal supporting program) is recommended to improve HRQoL among women with breast cancer in the post-surgery period.

## Data Availability

All data generated or analyzed during this study are included in this manuscript.

## Abbreviations

AGFI: adjusted goodness-of-fit index
BCS: breast-cancer-specific additional concerns
BI: Body image
BI-AC: BI-related arm change
BI-BC: BI-related behavior change
BI-SAC: BI-related sexual activity change
BI-SC: BI-related social change
BI-SCo: BI-related self-cognition
BI-PC: BI-related psychological change
BI-RC: BI-related role change
BISQ-BC: Body Image Self-Rating Questionnaire for Breast Cancer
BP: bodily pain
EWB: emotional well-being
FACT-Bv4.0: Functional Assessment of Cancer Therapy-Breast version 4.0
FWB: Functional well-being
GFI: Goodness-of-fit index
GH: General health
HRQoL: Health-related quality of life
INF: Information needs
MCS: Mental component summary
MH: Mental health
NC: Normed chi-square
NI: Needs importance
NS: Needs satisfaction
NSQ-BC: Needs Self-Rating Questionnaire for Breast Cancer
PCS: Physical component summary
PF: Physical function
PHY: Physical needs
PSY: Psychological needs
PWB: Physical well-being
RE: Role-emotional
REH: Rehabilitation needs
RMSEA: Root mean square error of approximation
RP: Role-physical
RSE: Respect/self-esteem needs
SEM: Structural equation modeling
SF: Social function
SF-36v2: 36-item Short Form Health Survey version 2
SWB: Social/family well-being
VT: Vitality
